# Impact of Endophytic and Rhizospheric Fungi on The Growth and Specialized Metabolite Production of *Phyllanthus niruri* L.

**DOI:** 10.21315/tlsr2025.36.2.13

**Published:** 2025-07-31

**Authors:** Winda Nawfetrias, Yuda Purwana Roswanjaya, Nur Alfi Saryanah, Sulastri Sulastri, Lukita Devy, Rizkita Rachmi Esyanti, Ahmad Faizal

**Affiliations:** 1 Plant Science and Biotechnology Research Group, School of Life Sciences and Technology, Institut Teknologi Bandung, Jl. Ganesa No. 10, 40132 Bandung, Indonesia; 2 Research Centre for Horticulture, Research Organization for Agriculture and Food, National Research and Innovation Agency (BRIN), KST Soekarno, Jl. Raya Jakarta-Bogor KM 46, Cibinong, 16915 Bogor, Indonesia; 3 Research Centre for Applied Microbiology, Research Organization for Life Sciences and Environment, National Research and Innovation Agency (BRIN), KST Soekarno, Jl. Raya Jakarta-Bogor KM 46, Cibinong, 16915 Bogor, Indonesia

**Keywords:** Elicitation, Endophytic Fungi, Lignan, *Phyllanthus niruri*

## Abstract

Medicinal plants are rich sources of specialised metabolites, crucial for various fields like agriculture, forestry, food processing, biofuels and environmental remediation. Microbes, particularly endophytic and rhizospheric fungi, play a significant role in optimising the production and regulation of these compounds. Several research showed these fungi in various plants, but they have not been thoroughly studied in *Phyllanthus niruri*, especially to promote growth and elicit lignan compounds like phyllanthin in *P. niruri*. A total of 131 fungi, consist of 57 rhizospheric fungi and 74 endophytic fungi, were successfully isolated from *P. niruri* in six different lowland areas in West Java. The potency assay results indicated that 106 fungi could produce indole-3-acetic acid (IAA). Six fungi could synthesise cellulase, and one fungus had the capability to solubilise phosphate. Our results showed that *Fusarium sp., Colletotrichum gloeosporioides, Colletotrichum tenuissimum, Colletotrichum fructicola, Pseudallescheria boydii, Aspergillus aculeatus, Myrothecium inundatum, Colletotrichum truncatum* can synthesise IAA. *Fusarium* sp. and *Myrothecium inundatum* could synthesise cellulase and only *Aspergillus aculeatus* have activity as phosphate solubilisation. Cocultivation of *P. niruri* and eight endophytic and rhizospheric fungi showed that *Fusarium* sp., *C. gloeosporioides, P. boydii, A. aculeatus* and *M. inundatum* had the potential traits to increase biomass, phyllanthin levels and phyllanthin yield. In general, these fungi have the potency to be elicitors to enhance phyllanthin in *P. niruri*.

**Highlights**

- A diverse community of 131 fungi, including 57 rhizospheric and 74 endophytic strains, was successfully isolated from *Phyllanthus niruri* in West Java.
- Our study found that several isolated fungi showed potential to enhance *Phyllanthus niruri* growth by producing indole-3-acetic acid, synthesising cellulase and showing phosphate solubilisation.
- *Fusarium* sp., *Colletotrichum gloeosporioides, Pseudallescheria boydii, Aspergillus aculeatus* and *Myrothecium inundatum* significantly increased biomass, *phyllanthin* levels and yield in *Phyllanthus niruri*.

## INTRODUCTION

During the COVID-19 pandemic in Indonesia, *Phyllanthus niruri*, also known as Meniran, was extensively utilised for producing phytopharmaceutical products like immunostimulants. Remarkably, compounds such as phylantusin C, phyllantin, hypophilantin and formononetin-7-O-glucuronide produced by this plant, demonstrated potential in binding SARS-CoV-2 proteins, as evidenced by molecular docking analysis (Hadi 2021; Murugesan *et al.* 2021; Rathinavel *et al.* 2020). Recognised as a medicinal plant with diverse pharmaceutical effects, *P. niruri* harbours endophytic and rhizospheric fungi. These fungal symbionts engage in metabolic interactions with the host plant, contributing to the biosynthesis of various bioactive compounds, including phyllanthin. Endophytic fungi from *P. amarus* are capable of producing phyllanthin (Kandavel & Sekar 2015), however, there is no information on endophytic fungi related to phyllanthin synthesis in *P. niruri*. This natural polyphenolic compound, belonging to the lignan family, is synthesised from aromatic protein amino acids such as phenylalanine and is involved in plant growth and pathogen defense. In Indonesia, phyllanthin has been employed as an indicator of herbal extract quality. Numerous studies have highlighted the pharmaceutical activity of phyllanthin, including hepatoprotective, antiviral, antioxidant and blood vessel tension modulation properties (Al Zarzour *et al.* 2017; Inchoo *et al.* 2011; Marhaeny *et al.* 2021; Thakur *et al.* 2014). Classified as dihydoxy-dibenzylbutane within the lignan group (Umezawa 2003), phyllanthin is hypothesised to share a biosynthetic pathway with secoisolariciresinol, owing to their structural similarities (Banerjee & Chattopadhyay 2010; Mazumdar & Chattopadhyay 2016). Although lignans in plants primarily hypothesised to defend against herbivores and pathogenic microbes, their exact biological functions remain not completely understood (Markulin *et al.* 2019). In response to fungal infections, certain plant species synthesise lignans, which act as phytoalexins (Gang *et al.* 1999) and play an important role in stress responses (Corbin *et al.* 2013). Fungal elicitation treatments have been shown to increase lignan synthesis in plants (Tashackori *et al.* 2018). Furthermore, endophytic fungi associated with *P. niruri* have demonstrated significant antifungal, cytotoxic and antioxidant activities (Hannana *et al.* 2020).

Endophytic fungi, by colonising internal plant tissues, improve plant growth, yield and active metabolite accumulation, while rhizospheric fungi significantly influence plant-microbe interactions (Khalil *et al.* 2021; Taghinasab & Jabaji 2020). Endophytic fungi isolated from medicinal plants have demonstrated various growth-promoting activities, including the secretion of extracellular lytic enzymes and the suppression of pathogenic microbes (Khalil *et al.* 2021). These endophytes hold the potential to transform agricultural practices by not only improving plant health but also by producing specialised metabolites. Interestingly, study case at *Cannabis sativa*, endophytic microorganisms are capable of producing compounds similar to those found in their host plants, paving the way for the synthesis of medicinal compounds (Taghinasab & Jabaji 2020). The root colonisation process, a critical phase in establishing plant-microbe symbiosis, involves complex chemical signalling in the rhizosphere. This includes exudation, interactions between plants and microbes, and between different microbes (Chagas *et al.* 2018). Fungi isolated from the rhizosphere have shown antagonistic activity against fungal pathogens, contributing to significant biocontrol efficacy, and have been observed to enhance plant growth and seedling emergence (Zohair *et al.* 2018). Endophytes produce diverse metabolites to compete with other microbes in the rhizosphere and among themselves, producing different bioactive compounds in various host organisms depending on specific requirements (Sharma *et al.* 2023).

Moreover, research indicates a strong correlation between the abundance of endophytic fungi and the biological activity of host plants. This interaction enhances plant survival in dynamic environments and regulates the production of bioactive compounds with medicinal properties (Caruso *et al.* 2020; Chen *et al.* 2021). Additionally, the use of *P. niruri* during the COVID-19 pandemic in Indonesia underscores its pharmaceutical relevance, with compounds like phyllanthin showing promise in molecular docking analysis against SARS-CoV-2 proteins (Hadi 2021; Murugesan *et al.* 2021; Rathinavel *et al.* 2020).

However, studies on the plant growth-promoting and lignan-elicitation activities of fungi associated with *P. niruri* are limited. This study hypothesises that endophytic and rhizospheric fungi from *P. niruri* are potent bioinoculants for promoting growth and eliciting phyllanthin, a key lignan. We focus on isolating and experimentally examining these fungi, assessing their impact on plant growth through IAA production, cellulase production and phosphate solubilisation. Additionally, we investigate the fungi’s capacity to elicit phyllanthin, exploring how plant-fungal interactions activate defense-related biosynthetic pathways to boost phytochemical accumulation.

This article aims to provide a comprehensive overview of the current knowledge regarding symbiotic relationships and specialised metabolite production between *P. niruri* and its associated fungi. We explored the mechanisms by which endophytic and rhizospheric fungi influence the growth and specialised metabolite production in *P. niruri*. The study underscores the importance of microbial partners in optimising the medicinal properties of plants. It opens avenues for sustainable approaches in enhancing the yield and quality of pharmacologically important compounds.

## MATERIALS AND METHODS

### Sample Collection

Healthy *P. niruri* were collected from six different ecological niches in West Java and Banten Province where they thrived amidst competitive flora, including weeds and other plant species. Samples were identified morphologically and collected in sterile plastic bags, labelled and transferred to the laboratory in an ice box for further analysis.

### Isolation of Fungi

#### Isolation of endophytic fungi

The isolation process was adapted from the procedure outlined by Turbat *et al.* (2020) with some modifications. Leaves, stems and roots were rinsed under tap water to eliminate surface debris and sand particles. Subsequently, they were surface-sterilised by soaking in 70% ethanol for 30 s, followed by immersing in a 2.5% sodium hypochlorite solution with Tween-20 for 3 min. After sterilisation, the samples were thoroughly rinsed with sterile distilled water and air-dried on sterile filter paper to remove excess moisture. The leaves, stems and roots were then cut into segments of approximately 0.5 cm using a sterilised scalpel. To prevent bacterial contamination, these segments were placed on Petri dishes containing potato dextrose agar (PDA) media, supplemented with 10 mg/L chloramphenicol. The dishes were sealed with parafilm and incubated at 27 ± 2°C under dark conditions for 1 to 2 weeks. During this period, the segments were periodically examined for fungal growth. Fungal colonies from the segments were transferred to new Petri dishes containing PDA to facilitate identification and purification purposes.

#### Isolation of rhizospheric fungi

The dilution method was used for isolating the rhizospheric fungi (Miao *et al.* 2016). Ten grams of the soil adhering to the roots were shaken off and mixed with 90 mL of sterile water in a conical flask. The mixture was shaken for 1 h in a shaker incubator, then serially diluted from 10^1^ to 10 ^3^ . To prevent bacterial growth, 0.1 mL of the 10^3^ dilution soil solution was plated on PDA with 10 mg/L chloramphenicol. Each plate was incubated at room temperature for a week or until mycelial growth was observable. At this point, emerging hyphae were transferred to sterile PDA plates for identification and purification.

### Potency Assays

#### Synthesis of Indole-3-Acetic Acid

The synthesis of indole-3-acetic acid (IAA) was analysed to investigate the multifunctional potential of the fungi, following the method of Junaidi and Bolhassan (2017). A 1 cm diameter fungal disc was inoculated into 15 mL of potato dextrose broth (PDB) supplemented with 0.5 g/L of tryptophan and incubated at 27 ± 2°C for 7 days. The culture suspension was then centrifuged at 3,220 g for 10 min. To the 1 mL supernatant, 1 mL of Salkowski’s reagent was added (comprising 1 mL 0.5 FeCl_3_ , 50 mL distilled water and 30 mL concentrated H_2_SO_4_) and incubated at room temperature for 30 min. A colour change from colourless to pink or red indicated the secretion of IAA by fungal endophytes in the extract. The concentration of IAA secreted by potential endophytes was quantified using a spectrophotometer at 540 nm, with the IAA concentration of each potential fungal strain determined using a calibration curve equation with commercial IAA (Merck, Germany, Cat: 1.00353.0010).

#### Phosphate solubilisation

Phosphate solubilisation capability was qualitatively assessed according to the method of Jasim *et al.* (2013). Isolates were inoculated onto Pikovskaya agar medium and incubated at 27 ± 2°C for 7 days. A control was set up using Pikovskaya agar medium without any isolate. The formation of a clear zone around the colony, resulting from the utilisation of tricalcium phosphate, was measured to evaluate the isolates’ ability to solubilise phosphate.

#### Production of cellulase

The isolates were screened for cellulolytic activity by inoculation into an agar medium containing carboxymethylcellulose (CMC), as described by Gupta *et al.* (2012). After incubation for five days at 27 ± 2°C, the plates were flooded with 0.1% Congo red solution for 20 min–30 min, then destained with 1 M NaCl for 15 min to remove excess Congo red solution. The presence of a clear zone around the colour was indicative of cellulase-positive (Gupta *et al.* 2012).

#### Molecular Identification

Eight selected fungi that have potency as IAA producers, cellulase producers or phosphate solubilisation activity were grown in PDB supplemented with 10 mg/L chloramphenicol and incubated at 27 ± 2°C. After 7 days, the inoculum was filtered using sterile gauze. A total of 50 mg of mycelium was ground using liquid nitrogen, and DNA was extracted using Geneaid DNA Mini Kit (Geneaid Biotech Ltd. Cat: GP100). The DNA was amplified using ITS 1 primer (White *et al.* 1990) in Extragene EG9600 Gradient Thermocycler Polymerase Chain Reaction (PCR). The PCR mixture (50 μL) consisted of MyTaqTM HS Red Mix Bioline (Meridian Bioscience, CAT: BIO-25048), 10 pmol of each primer, ddH_2_O, and template DNA. The PCR conditions were as follows: Initial denaturation at 95°C for 3 min, followed by 35 cycles of denaturation at 95°C for 30 s, annealing at 52.5°C for 30 s and extension at 72°C for 30 s, with a final extension at 72°C for 10 min. The PCR product was visualised using 0.8% agarose gel on the Mupid Exu Electrophoresis System at 100 volts for 40 min. Sequencing was performed using Sanger sequencing on an ABI PRISM 3730xl Genetic Analyser by Applied Biosystems, USA. Sequence data processing was carried out by aligning with the Basic Local Alignment Search Tool (BLAST) against the public database reference genome from the National Centre for Biotechnology Information (NCBI) (http://www.ncbi.nlm.niv.gov). This alignment was performed to identify similarities between test sequences and those in the database.

### Application of Fungi as Growth Plant Promotor and Elicitor

#### Assessment of fungi application on plant

The experiment was divided into tray and pot segments. The experiment was conducted to examine the effect of eight selected fungi on the growth and phyllanthin content of *P. niruri*. The eight fungi were selected based on their ability to synthesise IAA, synthesise cellulase and have activity as phosphate solubilisation. The eight selected fungi were first cultured on PDA medium, and incubated at 27 ± 2°C in dark conditions for 7 days. Each plate was filled with 10 mL of sterile water, and spores were gently scraped off the medium’s surface using a sterile spreader to measure the quantity of spores (Zhu *et al.* 2014). The spore density was adjusted by a hemocytometer and light microscope (100× magnification). A spore density of 1 × 10^10^ cfu/mL was used in the experiments. The inoculum was inoculated into a 1:1 mixture of sterile soil and compost (approximately 1 kg, autoclaved at 121°C for 15 min) in 12 cm × 25 cm trays. For the pot experiment, the fungi were grown in PDB medium, incubated at 25°C with shaking for seven days and subsequently inoculated into soil:compost:husk (1:1:1) (approximately 1.5 kg, autoclaved at 121°C for 15 min) in 25 cm × 25 cm pots. The spore density was adjusted by a hemocytometer and light microscope (100× magnifications). A spore density of 1 × 10^10^ cfu/mL was used in the experiments. The experiments used the seeds of *P. niruri* collected from Jember, West Java, Indonesia. For each experiment, 2 g of seeds for the tray and 3 g for the pot were sterilised before use. Seeds were surface sterilised by immersion in 70% alcohol for 30 s, followed by sodium hypochlorite (5.25% active chlorine) with three drops of Tween 20 added for 20 min. The seeds were rinsed three times with sterilised (121°C for 15 min) deionised water for 1 min to remove all traces of the sterilant. Growth parameters observed included shoot length, root length, root shoot ratio, fresh weight and dry weight. Chlorophyll content was analysed using a soil-plant analysis development (SPAD) meter (Chlorophyll meter SPAD-502 Plus, Konica Minolta), which quantified the relative amount of chlorophyll by measuring the absorbance at 400 nm–500 nm and 600 nm–700 nm, providing a reading in arbitrary units proportional to the leaf chlorophyll content (Loh *et al.* 2002).

#### Phyllanthin extraction and analysis

The phyllanthin content was measured following the method described by Murugaiyah and Chan (2007) with modification. Aerial parts of *P. niruri* were dried at 40°C for 7 days and then pulverised before analysis. Phyllanthin standards of approximately 98.9% purity were purchased from Sigma Aldrich, Singapore (CAT: 1536509). All solvents used for the analysis were obtained from Merck (Darmstadt, Germany). For extraction, 250 mg of dry powder *P. niruri* was dissolved in 25 mL methanol and subjected to sonication at 45°C for 30 min. The extracts were incubated overnight at 25°C, filtered by Whatman filter paper, and evaporated at 20°C, 100 rpm. The residue was diluted with acetonitrile:deionised water (55:45 v/v) until the final concentration was 20 mg/mL–50 mg/mL. Both standards and extracts were filtered through a 0.45 μm nylon membrane filter obtained from Whatman (Kent, UK). Subsequent filtration of the samples was conducted using a 0.45 μm PTFE syringe filter (Whatman, Maidstone, England). For high-performance liquid chromatography (HPLC) analysis, a 10 μL sample was injected into the HPLC (Waters with 2695 separation module) coupled to a fluorometric detector (FD Waters multi fluorescence detector 2475), and 2998 PDA detector. A Mastro2 C18 column (100 mm × 2,0 mm × 3 μm) was used for chromatographic separation. The column temperature was set at 35 ± 1°C. The mobile phase used is acetonitrile:water (55:45 v/v), with a flow rate of 1 mL/min at room temperature (25°C). The analysis time is as follows: 30 min of preparation, 30 min of analysis time and 96 min of column washing. The chromatogram is observed at a wavelength of 344 nm. The concentration of phyllanthin in the sample is calculated based on the linearity of its chromatography to the standard curve. The level of phyllanthin is calculated by multiplying the ratio of the sample area to the standard area by the standard concentration and then further multiplying this result by the ratio of the sample volume to the sample volume weight. The yield of phyllanthin is determined by multiplying dry weight and level of phyllanthin.

### Statistical Analysis

All statistical analyses, including determining significant differences, were carried out using R statistical software version 4.3.3. A one-way ANOVA, followed by Duncan’s multiple comparisons (*p*-values < 0.05), was used to assess the significant differences among various cocultivation treatments. Principal component analysis (PCA) was utilised to analyse the distribution patterns of cocultivation treatment based on the observed variables.

## RESULTS

### Exploration and Isolation of Endophytic and Rhizospheric Fungi

Fungal exploration was carried out in six lowland areas of West Java (Table 1). A total of 131 fungi, comprising 57 rhizospheric fungi and 74 endophytic fungi, were isolated from *P. niruri*, which thrives as a weed or shrub in diverse habitats, including rocky soil and topsoil.

**Figure 1:**
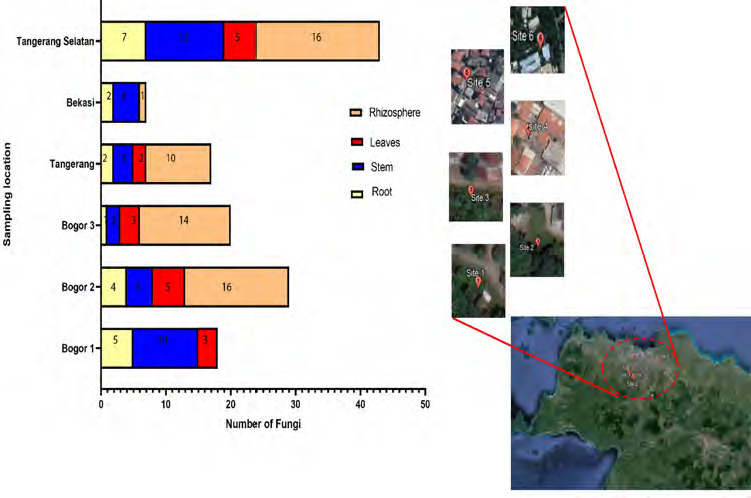
Abundance of endophytic and rhizospheric fungi in West Java and Banten Province. Site 1: Bogor 1; Site 2: Bogor 2; Site 3: Bogor 3; Site 4: Tangerang; Site 5: Bekasi; Site 6: Tangerang Selatan. Bogor 1 and Bekasi: rocky soil. Bogor 2, Bogor 3, Tangerang, Tangerang Selatan: topsoil (*Source:* Google Earth Engine).

The fungi were successfully isolated from the roots (15 fungi), stems (21 fungi), leaves (14 fungi) and rhizospheric areas (56 fungi) of *P. niruri* growing in topsoil (Tangerang Selatan, Tangerang, Bogor 3, Bogor 2). Fungi were not optimally isolated from rocky soil in Bekasi and Bogor 1, as endophytic fungi could not be found in leaf tissues (Bekasi) or the rhizosphere (Bogor 1). The fungi isolated from the roots, stem, leaves and rhizospheric area at rocky soil are 7, 14, 3 and 1 fungi, respectively. Endophytic and rhizospheric fungal abundance in *P. niruri* was lower in rocky soil habitats compared to topsoil habitats, with successful isolation rates varying across plant tissues and locations (Fig. 1).

### Potency Assay of Endophytic and Rhizospheric Fungi

**Figure 2:**
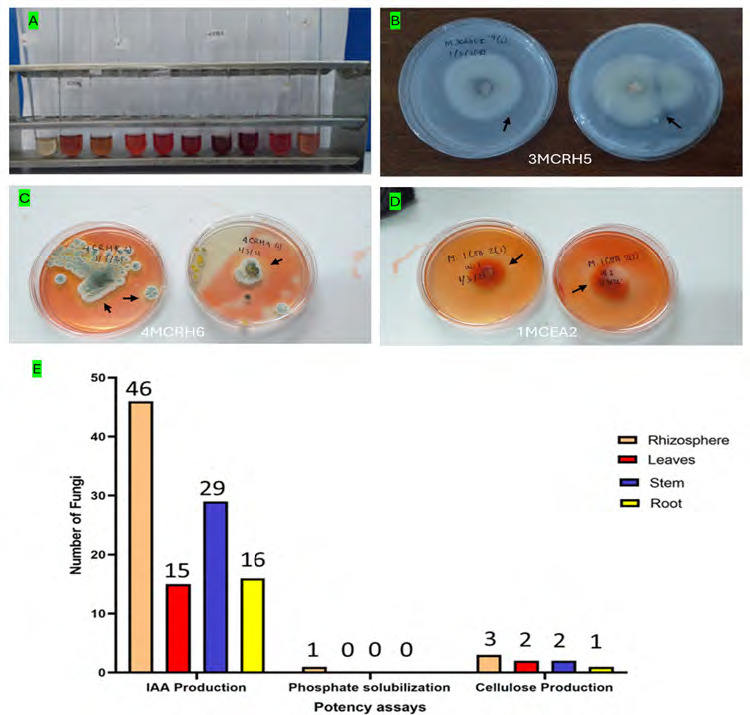
Example of potency assays for (A) IAA synthesis, (B) phosphate solubilisation and (C, D) cellulase synthesis. (E) The assay result of endophytic and rhizospheric fungi of *P. niruri*.

The total of 131 fungi, consisting of 57 rhizospheric fungi and 74 endophytic fungi, were explored and tested for their ability to synthesise IAA, cellulase and solubilise phosphate. The production of IAA was detected using Salkowski’s method, with pink or red colour changes in the extract after incubation indicating the IAA secret (Fig. 2A). Phosphate solubilisation activity was evidenced by the formation of a clear zone (indicated by a black arrow in the figure) around the fungal colony on Pikovskaya medium containing tricalcium phosphate (Fig. 2B). The capacity to synthesise cellulase was demonstrated by clear zones on the medium containing CMC, indicated by black arrow in Figs. 2C and 2D. The assay results indicated that 106 fungi could produce IAA, 8 had the potential to synthesise cellulase and 1 fungus had the capability to solubilise phosphate (Fig. 2E).

**Figure 3:**
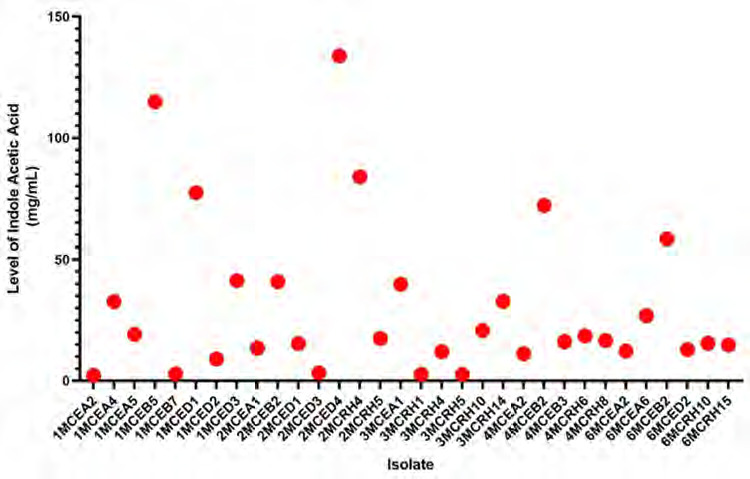
The potency of IAA synthesis in endophytic and rhizospheric fungi of *P. niruri*.

Among these, 32 fungi could produce more than 2 mg/mL of IAA (Fig. 3). The concentration of IAA produced by these fungi ranged from 2.24 mg/mL to 133.77 mg/mL. Notably, six fungi demonstrated higher IAA than others, such as 1MCEB5, 1MCED1, 2MCED4, 2MCRH4, 4MCEB2 and 6MCEB2, with concentrations of 114.85 mg/mL, 77.362 mg/mL, 133.77 mg/mL, 83.856 mg/mL, 72.08 mg/mL and 58.316 mg/mL, respectively. These fungi were selected for further experiments in planta.

The potency test also revealed that five fungi could synthesise cellulases, specifically 1MCEA2, 1MCEB7, 1MCED1, 1MCED2 and 4MCRH6. These fungi exhibited clear zones on the CMC selective medium, utilising the cellulose in CMC media as a carbon source for growth. In the phosphate solubilisation activity test conducted on the Pikovskaya selective medium, only 3MRH5 was detected as capable of solubilising phosphate, as indicated by a clear zone in the medium. The eight fungi with the highest IAA synthesis, with phosphate solubilising potency and could produce cellulase were identified through molecular methods and selected for subsequent experiments in the planta. Fungi associated with *P. niruri* are primarily capable of synthesising IAA, with higher occurrence in the rhizosphere and stems.

### Molecular Identification

DNA from eight selected fungi, consisting of five endophytic fungi and three rhizospheric fungi, was amplified using the ITS1 primer, yielding a PCR product of approximately 600 bp. Sequence analysis putatively identified these eight fungi as distinct species. *Fusarium* sp. was isolated from roots; *Colletotrichum gloeosporioides* and *Colletotrichum truncatum* were isolated from stems; *Cladosporium tenuissimum*, *Colletotrichum fructicola* were isolated from leaves; *Pseudallescheri boydii*, *Aspergillus aculeatus* and *Myrothecium inundatum* were isolated from the rhizosphere (Table 2).

### Assessment of Fungi Application on Plant

#### Tray experiment

**Figure 4:**
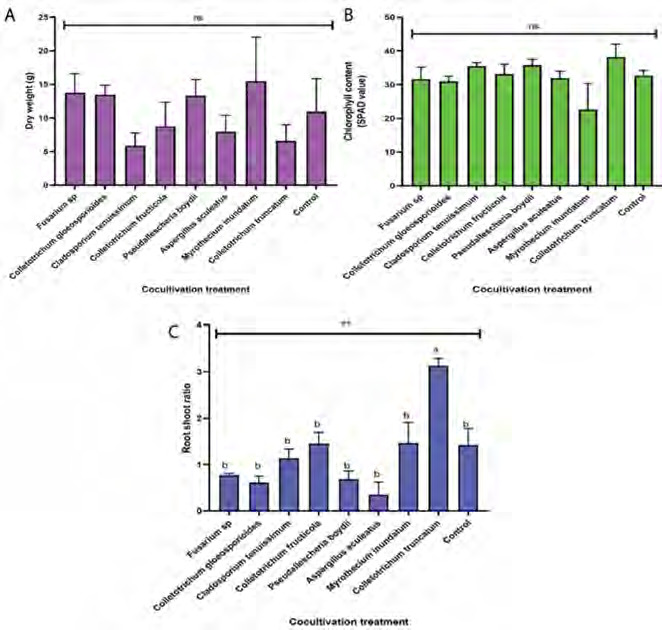
Effect of fungal cocultivation on *P. niruri* in trays experiment: (A) dry weight, (B) chlorophyll content and (C) root shoot ratio. Means with different letters were significantly different at *p* < 0.05.

The result of the ANOVA for fungi application showed the non-significant effect of the dry weight (*p* = 0.681) on the tray experiment at *p* < 0.05 (Fig. 4A), suggesting that selective fungi cocultivation did not exhibit pathogenic symptoms or adversely affect plant growth. The total chlorophyll level was also similar across all cocultivation treatments compared to the control (*p* = 0.172) (Fig. 4B), indicating no impact on the photosynthesis process and consequently, on dry weight. Cocultivation of *C. truncatum* resulted in the highest root-shoot ratio based on Duncan’s multiple comparisons (*p* < 0.05), significantly differing from other treatments and the control (*p* = 0.001), suggesting a greater biomass allocation to roots (Fig. 4C).

**Figure 5:**
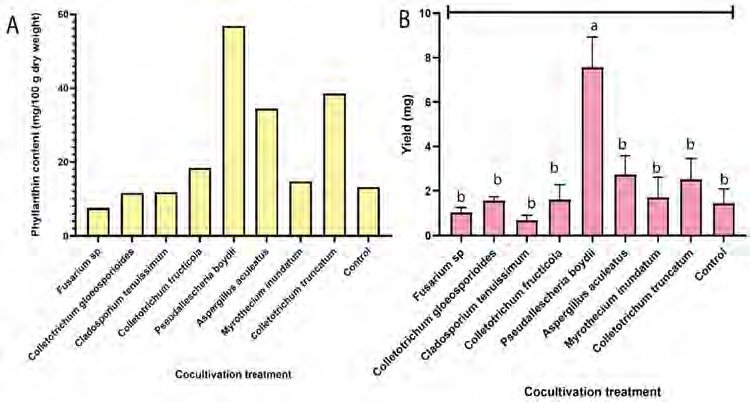
Effect of fungal cocultivation on *P. niruri* in tray experiment: (A) phyllanthin levels and (B) yield of phyllanthin. Means with different letters were significantly different at *p* < 0.05.

Phyllanthin level is determined by composting the extracts from all repetitions for each treatment. Cocultivation with *C. fructicola, P. boydii*, *A. aculeatus*, *M. inundatum* and *C. truncatum* resulted in higher phyllanthin levels compared to the control (Fig. 5A). Cocultivation of *P. boydii* showed higher phyllanthin yield (*p* = 0.001) compared to the control (Fig. 5B).

**Figure 6:**
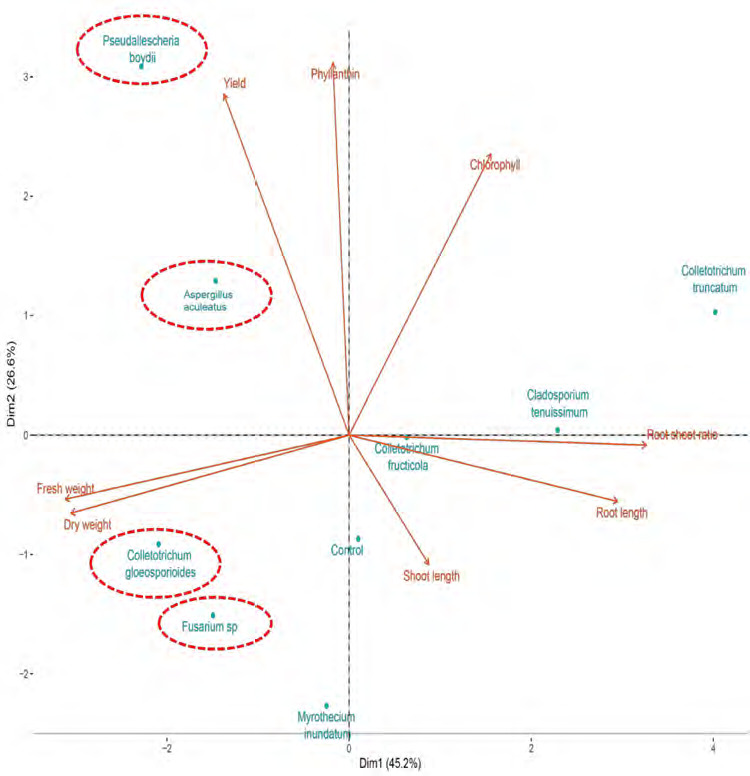
PCA of growth parameters, phyllanthin levels and yield of phyllanthin in cocultivation treatment on *P. niruri* in trays experiment.

PCA highlighted the relationships between observed variables and cocultivation treatments. The first two components explained 71.8% of the variation, with PC1 accounting for 45.2% and PC2 for 26.6% (Fig. 6). Variables were categorised into four quadrants: Chlorophyll level in quadrant I, phyllanthin level and phyllanthin yield in quadrant II, fresh and dry weight in quadrant III, and root shoot ratio, root length and shoot length in quadrant IV. The grouping of fungal species in these quadrants indicated their specific influences on *P. niruri* growth parameters and phyllantin production. PCA reveals *P. boydii* and *A. aculeatus* tend to increase phyllanthin levels and yield, while *C. gloeosporioides* and *Fusarium* sp. tend to increase fresh and dry weight of *P. niruri*.

#### Pot experiment

**Figure 7:**
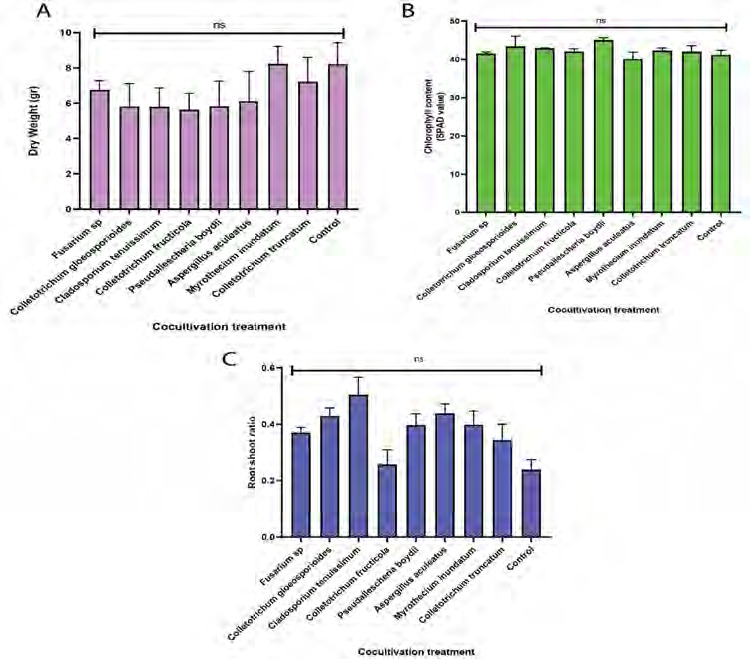
Effect of fungal cocultivation on *P. niruri* in a pot experiment regarding (A) dry weight, (B) chlorophyll content and (C) root shoot ratio. Means with different letters were significantly different at *p* < 0.05.

Similar to the tray experiment, the pot experiment showed no significant differences in dry weight (*p* = 0.549) and total chlorophyll levels (*p* = 0.574) between the cocultivation treatment and control (Figs. 7A and 7B), reinforcing that the selected fungi did not affect chlorophyll synthesis or photosynthesis process. Cocultivation of *C. tenuissimum* resulted in the highest root-shoot ratio based on Duncan’s multiple comparisons (*p* < 0.05), significantly differing from other treatments and the control (*p* = 0.012), suggesting a greater biomass allocation to roots (Fig. 7C).

**Figure 8:**
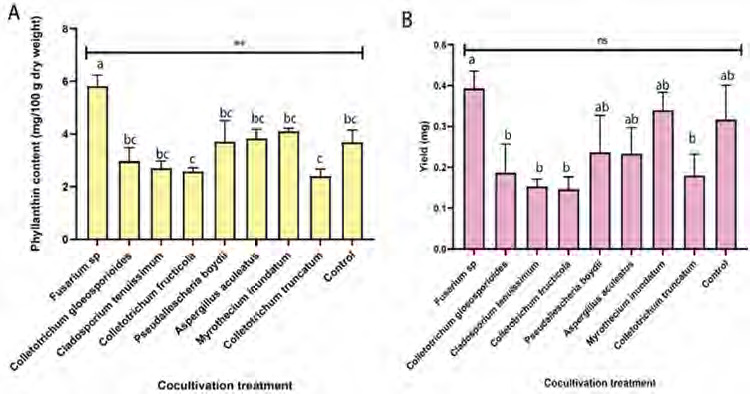
The impact of fungal cocultivation on *P. niruri* in pot experiment on (A) phyllanthin levels and (B) yield of phyllanthin. Means with different letters were significantly different at *p* < 0.05.

Cocultivation treatment significantly affected phyllanthin levels (*p* = 0.001) but not phyllanthin yield (*p* = 0.089) (Fig. 8). *Fusarium* sp. exhibited markedly higher phyllanthin levels in comparison to other treatments.

**Figure 9:**
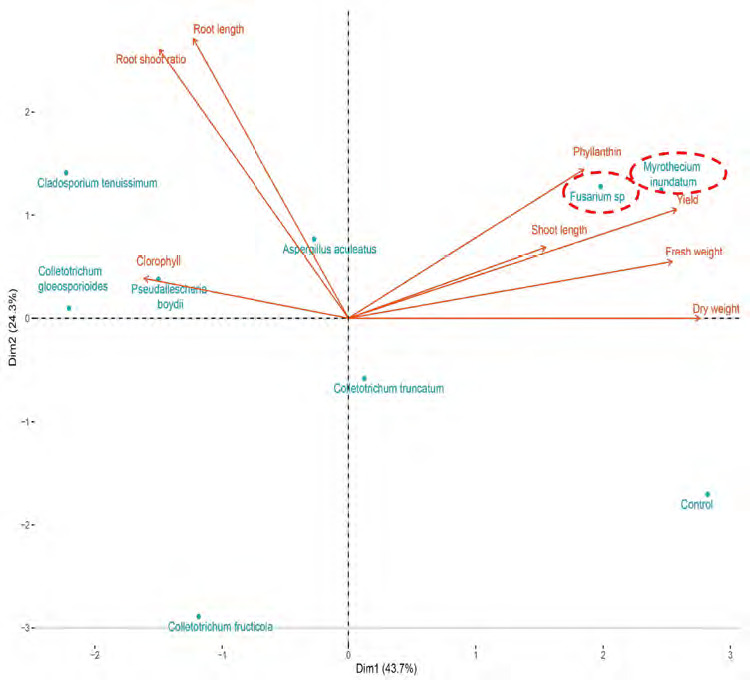
PCA of growth parameters, phyllanthin levels and yield of phyllanthin under cocultivation treatment on *P. niruri* in pot experiment.

The first two components accounted for 68.0% variations, with PC1 at 43.7% and PC2 at 24.3% (Fig. 9). The variables are distributed across four quadrants in the loading plot. Quadrant I include fresh weight, dry weight, shoot length, phyllanthin level and yield. Quadrant II comprises root length, root shoot ratio and chlorophyll level. The third and fourth quadrants did not display any observed variables. Cocultivation treatment grouped *Fusarium* sp. and *M. inundatum* in quadrant I, *A. aculeatus*, *P. boydii*, *C. tenuissimum* and *C. gloeosporioides* in quadrant II, *C. fructicola* in quadrant III and *C. truncatum* in quadrant IV. These associations suggest that cocultivation with *Fusarium* sp. and *M. inundatum* enhances the phyllanthin level, yield, shoot length, fresh weight and dry weight of *P. niruri*. Cocultivation of *A. aculeatus* and *C. tenuissimum* increases root length and root-to-shoot ratio, while cocultivation of *P. boydii* and *C. gloeosporioides* boosts chlorophyll levels. Cocultivation with *C. truncatum*, *C. fructicola*, as well as the control did not affect the observed variables.

## DISCUSSION

Topsoil habitats exhibited a higher abundance of endophytic and rhizospheric fungi compared to rocky soil habitats, suggesting that soil type influences the prevalence of these fungi in *P. niruri* tissues (Table 1; Fig. 1). The abundance of fungi in different soil habitats, such as topsoil versus rocky soil, is influenced by factors such as nutrition content, water level and soil texture. These factors, along with the overall ecosystem, host plant cultivars, geographic location and soil type, contribute to the diversity and abundance of microorganisms. Factors such as soil type, plant tissue type and habitat also play a role in shaping the microbial community structure (Blodgett *et al.* 2007; Eschen *et al.* 2010; Lu *et al.* 2016; Pili *et al.* 2016; Wu *et al.* 2013; Xia *et al.* 2020).

Our study revealed that the number of fungi successfully isolated from the rhizosphere was higher than from plant tissues (leaves, stems and roots) (Fig. 2). This is because the plant organ or phyllosphere is a short-lived environment, as opposed to the rhizosphere, which comprises the area in the soil around plant roots. Conversely, this result aligns with the fact that the rhizosphere is a critical zone for microbial interactions, where exudates can influence fungal populations and serve as a battleground between beneficial and detrimental fungi (Raaijmakers *et al.* 2009). While this might suggest a higher fungal density in the rhizosphere, it is crucial to acknowledge that the number of isolates does not directly equate to actual fungal abundance. Several factors, including the suitability of culture media and the technical proficiency of isolation methods, can significantly influence the number of fungi recovered. Furthermore, among the plant tissues, stems exhibited the highest number of fungal isolates, followed by roots and then leaves (Fig. 2). This observation aligns with findings in other plant species, such as *Hevea brasiliensis* and rice, where stems often harbour greater fungal diversity. This might be attributed to the deposition of airborne spores on the stem surface and their subsequent colonisation (Araújo *et al.* 2020; Wang *et al.* 2016).

It’s important to note that various factors, including plant tissue type, plant growth stage and environmental conditions can influence the composition of the endophytic fungal community (Robinson *et al.* 2016). While this study provides insights into the distribution of fungi across different plant organs and the rhizosphere of *P. niruri*, further research is necessary to fully understand the factors driving fungal diversity and abundance in this plant-soil system.

The abundance of IAA-producing fungi varies across different plant parts, with the highest concentration found in the rhizosphere, followed by the stems, roots and leaves (Fig. 2). The rhizosphere, a thin layer of soil surrounding plant roots, is a crucial area for microorganism interaction and plant growth. Roots secrete organic compounds like sugars, acids and vitamins, which fungi use as nutrients or signals. Fungi release iron carriers, volatile compounds and plant hormones, which may enhance plant growth by increasing nutrient availability to their host (Fu *et al.* 2015; Huang *et al.* 2014; Saharan & Nehra 2011). Our study extends these findings, revealing that the IAA-producing fungi are present not only in the roots of *P. niruri* but in the stem and leaves, as well as in the rhizosphere. The production of IAA by endophytes, which reside inside the stems or leaves, may also aid the host in surviving in hostile environments (Khan *et al.* 2016).

Endophytic fungi, which produce hydrolytic enzymes like cellulase, are less common than IAA-producing fungi. These enzymes are important in the mutualistic relationship between endophytic fungi and the host plant, aiding in cellulose degradation. Fungi with cellulolytic enzymes can adapt to local environmental changes and compete with other organisms. Some fungi adopt a saprotrophic lifestyle, degrading plant cell wall cellulose, requiring key element identification for switching strategies (Strakowska *et al.* 2014; Moy *et al.* 2002). Phosphate-solubilising fungi were found exclusively in the rhizosphere. Commonly, this fungus is known to enhance plant growth by providing nutrients, producing biocontrol metabolites, protecting against pathogens and synthesising growth hormones, while also generating organic acids for acidic environments (Walia *et al.* 2017; Zhang *et al.* 2018). The study of cellulase-producing fungi and phosphate-solubilising fungi isolated from *P. niruri* is limited. Some research focuses on bacteria (Joe *et al.* 2016), while others show phosphate-solubilising fungi isolated from shoots on *P. niruri* (Bhatia *et al.* 2019).

Eight endophytic and rhizospheric fungi capable of synthesising IAA, cellulase and solubilising phosphate were identified by ITS 1. The putative fungi recognise as *Fusarium* sp. from roots, *C. gloeosporioides* and *C. truncatum* from stems, *C. tenuissimum* and *C. fructicola* from leaves, *P. boydii*, *A. aculeatus* and *M. inundatum* from the rhizosphere. These genera harbour both endophytic and rhizospheric species in various plant hosts. For instance, species of *Colletotrichum* can reside asymptomatically in weed species, with dormancy breaking as the plant matures (Ranathunge *et al.* 2016). The genera *Fusarium*, *Colletotrichum* and *Aspergillus* are common endophytes in *Phyllanthus*, with several species like *F. oxysporum*, *Alternaria* sp., *Gibberella moniliformis* (Synonym: *F. verticillioides*), *Edenia gomezpompae*, *C. circinans*, *C. crassipes*, *C. falcatum*, *C. truncatum*, *Phoma epicoccinia*, *P. chrysanthemicoli*, *Phyllosticta* sp., *Aspergillus nidulans*, *Penicillium citrinum*, *Pestalotiopsis* sp. identified in *P. amarus* tissue (Kandasamy *et al.* 2015; Kandavel & Sekar 2015), and *Curvularia geniculata* isolated from *P. niruri* tissue (Kalimuthu *et al.* 2022).

Our results indicated that *Fusarium* sp. isolated from *P. niruri* can synthesise both IAA and cellulase (Table 2). Similarly, *A. aculeatus* from *P. niruri* can produce IAA and solubilise phosphate, aligning with other findings that *Fusarium* and *Aspergillus* genera possess IAA synthesis capability (Bilal *et al.* 2018; Junaidi & Bolhassan 2017; Noorjahan *et al.* 2019; Al-Harthi *et al.* 2023). *M. inundatum* from *P. niruri* can synthesise IAA and cellulase, while *C. tenuissimum* is known only synthesis IAA. Fungi from genus *Fusarium*, *Aspergillus* and *Myrothecium* isolated from the root and rhizosphere of *P. niruri*, respectively, are identified as producing IAA. The root and rhizosphere of *P. niruri* provides suitable environment for fungi to produce IAA. *Fusarium delphinoides*, a fungal strain from chickpea rhizospheric areas, produces and secretes IAA *in vitro*, relying on tryptophan as a nitrogen source. Growth conditions, pH, tryptophan concentration and carbon source influence IAA production. Tryptophan serves as a precursor for auxin biosynthesis in plants and microbes, with the most efficient auxin producers found in plant rhizosphere and phyllosphere (Kulkarni *et al.* 2013; Chung *et al.* 2003; Spaepen & Vanderleyden 2011; Kravchenko *et al.* 2004; Tsavkelova *et al.* 2005). Our research also found fungi known as *Cladosporium* isolated from leaves of *P. niruri* that produced IAA. This finding aligns with Yang *et al.* (2023) that suggests *Cladosporium* as a novel endophytic fungus that promotes plant growth in both monocot and dicot plants. Its genome sequence reveals its ability to produce IAA, potentially enhancing plant growth through indirect effects on nitrogen uptake.

Generally, IAA synthesis is crucial for mycelial growth and pathogenic development. Fungal-produced IAA stimulates root formation, enhances nutrient absorption and bolsters plant immune response. It also acts as a signalling molecule in microorganisms, affecting gene expression. IAA synthesis by fungi regulates plant growth and facilitates symbiotic interactions between plants and microorganisms (Contreras-Cornejo *et al.* 2009; Dong *et al.* 2022; Junaidi & Bolhassan 2017; Lin & Xu 2013; Ludwig-Müller 2015; Yuan *et al.* 2008).

The cocultivation of these potential fungi with *P. niruri* showed no pathogenic symptoms in both the tray and pot environments. This finding suggests that the fungal density might be insufficient for colonisation of the *P. niruri* tissue. Furthermore, tray and pot environments do not cause these fungi to be pathogenic. Fungi pathogenicity depends on the density of fungal cells colonising host tissue, with some entering a latent phase and others facultative parasites. Endophytic fungi can become pathogenic under stress conditions (Compant *et al.* 2010; Photita *et al.* 2004).

Our experiment found that cocultivation did not affect the dry weight and chlorophyll level of *P. niruri*. The relationship between endophyte cocultivation and total chlorophyll concentration appears weak (Sanchez-Azofeifa *et al.* 2012). Similarly, the application of fungal elicitors in *Atractylodes lancea*, both exogenous and endophytic, had minimal effect and there was no significant difference in photosynthetic characteristics, such as chlorophyll levels, carbohydrate levels and photosynthetic rates compared to the controls (Wang *et al.* 2012). Cocultivation treatment resulted in a significantly different root shoot ratio, particularly with *C. truncatum*. This may suggest that inoculation with this fungus induces stress, prompting *P. niruri* to allocate more growth to the roots. A higher root biomass can signify the plant’s adaptability to stress and ability to maximise nutrient uptake (Shymanovich & Faeth 2019). Typically, plants have a root shoot ratio of 1:5 to 1:6 under normal conditions. A lower ratio may reflect better-growing conditions, while an increase in ratio could indicate growth under less favorable circumstances (Harris 1992). The *Colletotrichum*, known for causing anthracnose disease in various horticultural crops, can also produce various secondary metabolites and diverse bioactivities. Over 109 such metabolites from the *Colletotrichum* have been identified (Wha *et al.* 2019). Interestingly, *C. gloeosporioides* extracted from the lignan-rich flowering plant, *Forsythia suspensa*, has been reported to synthesise the lignan compound, phillyrin (Zhang *et al.* 2012).

Cocultivation with *C. fructicola, P. boydii*, *A. aculeatus*, *M. inundatum* and *C. truncatum* resulted in elevated phyllanthin levels in *P. niruri*, with only *P. boydii* showing a significant increase in phyllanthin yield compared to the control in tray experiment. Conversely, in the pot experiment, *Fusarium* sp. demonstrated a notable increase in phyllanthin levels, although its effect on phyllanthin did not differ from the control. PCA suggests that *P. boydii* and *A. aculeatus* tend to boost phylanthin levels and yield of phyllanthin, while *C. gloeosporioides* and *Fusarium* sp. are more inclined to enhance biomass in tray experiments. In pot experiments, however, *Fusarium* sp. and *M. inundatum* are inclined to augment biomass, phyllanthin level and yield. In general, *P. boydii*, *A. aculeatus*, *Fusarium* sp. and *M. inundatum* exhibit the potential to raise phylanthin levels and yield, whereas *C. gloeosporioides* appears to have the capacity to increase the biomass of *P. niruri*.

Endophyte fungi can enhance lignan synthesis in medicinal plants, improving *Phyllanthus* species by modulating secondary metabolite biosynthesis that crucial for human health and optimising production under *in vitro* and *in vivo* conditions (Nawfetrias *et al.* 2024). These observations imply that the synthesis of the lignan compound phyllanthin may be influenced by the interaction between the host plant and the associated fungi. Some fungi can synthesise the same compound as their plant hosts, as seen with endophytic fungi, *Aspergillus*, associated with *Juniperus*, and synthesise aryl tetralin lignans analogous to their host (Kusari *et al.* 2009). These compounds are crucial for the environmental defense mechanisms of the host plants. *Myrothecium* species, endophytic fungi isolated from *Calotropis procera*, have been identified as potential antioxidants, contributing to survival in stressful environments (Ahmed El-Khawaga *et al.* 2013).

The mutual relationship between plants and fungi is beneficial as fungi aid plant growth by producing growth-enhancing substances such as hormones, enzymes and other compounds. Yet, this interaction is influenced by specific genes and environmental conditions, causing variability in experimental assays. This inconsistency challenges the commercial use of fungi as stimulants. To overcome this, we recommend using these substances directly, rather than the organisms, to minimise environmental and genetic influences. This approach could be simpler and less equipment-intensive.

In light of concerns about harmful effects on agriculture chemicals, it is crucial to deepen our comprehension understanding on the role beneficial microbes in agriculture, particularly with medicinal plants. Plant-growth-promoting fungi are promising for sustainable farming due to their eco-friendly and cost-effective enhancement of plant growth and yield. However, their practical use is limited by variability in field performance, which is a major hurdle in the development of future progress of microbial inoculants for plant growth. As we gain more knowledge about the complex root ecosystem, the function of these fungi and the logistic of producing, formulating and applying these inoculants, the availability of beneficial fungi products will likely increase. Understanding different microbial interactions that offer diverse or complementary benefits is crucial for improving bio-formulation. Applying modern biotechnological methods and reliable transformation systems can help modify beneficial fungi to enhance their benefits to crops. Genetically enhancing specific growth-promoting traits could lead to more effective inoculants, particularly if these traits are synergistic. Regular research is needed to monitor the genetic stability and ecological impact of these genetically modified strains. Efforts should be intensified to enhance the connection between researchers and business owners to facilitate technology transfer and encourage adoption by end-users.

## CONCLUSION

Endophytic and rhizosperic fungi isolated from *P. niruri*. demonstrate the potential to synthesise IAA, cellulase and solubilise phosphate. Cocultivation experiment revealed that *Fusarium* sp., *C. gloeosporioides*, *P. boydii*, *A. aculeatus* and *M. inundatum* possess traits that potentially enhance biomass, as well as increase levels and yield of phyllanthin in *P. niruri*.

## Figures and Tables

**Table 1:** Locations of exploration of endophytic and rhizospheric fungi from *P. niruri.*

Location	Habitat	Altitude (m asl)	Longitude
Bogor 1, Gunung Sindur	Rocky soil	80.2	6°23′32.35″S;106°42′58.16″E
Bogor 2, Gunung Sindur	Topsoil	80.2	6°23′32.08″S;106°42′57.24″E
Bogor 3, Gunung Sindur	Topsoil	80.5	6°23′32.65″S;106°42′58.97″E
Tangerang, Karang Tengah	Topsoil	15.9	6°12′58.52″S;106°42′30.57″E
Bekasi, Bekasi Jaya	Rocky soil	15.2	6°13′44.75″S;107° 0′56.70″E
Tangerang Selatan, Setu	Topsoil	59.4	6°21′26.35″S;106°39′56.14″E

**Table 2:** Functional characteristics of fungi isolated from *P. Niruri*: IAA production, cellulase production and phosphate solubilisation.

Origin	% indent	Accession	Species	Fungi potency			Phenotype
				IAA prod.	Cellulase prod.	Phosphate solubiliser	
Roots	97.35	LC683321.1	*Fusarium* sp.	+	+	−	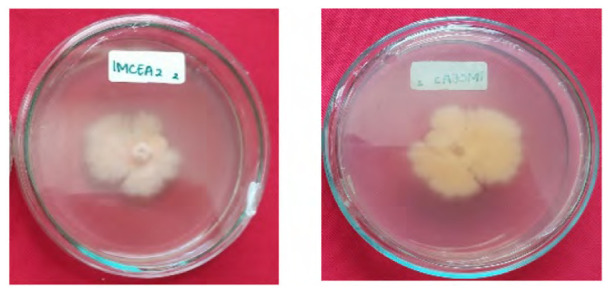
Stems	98.75	MK070140.1	*C. gloeosporioides*	+	−	−	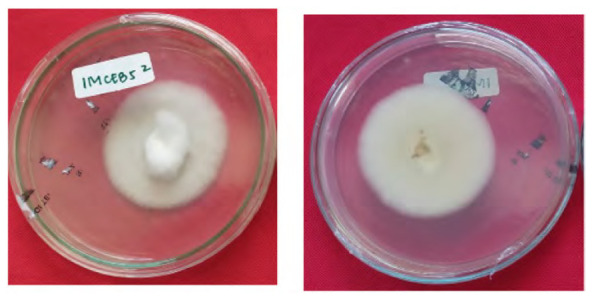
Leaves	99.04%	MF473304	*C. tenuissimum*	+	−	−	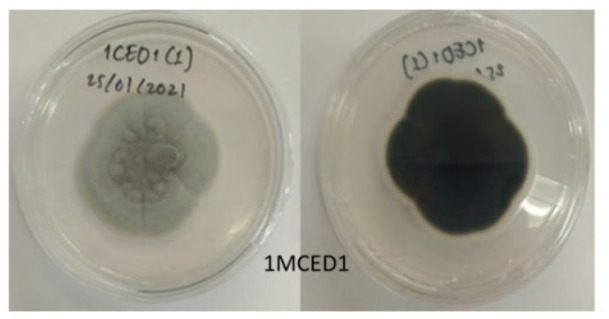
Leaves	99.34%	MT626035	C. fructicola	+	−	−	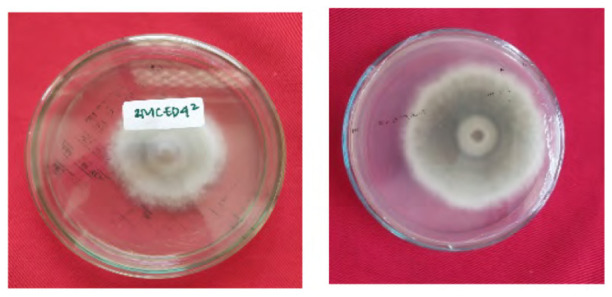
Rhizosphere	99.65	AY217658	P. boydii	+	−	−	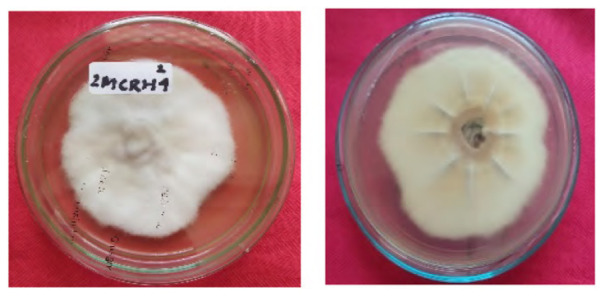
Rhizosphere	100.00	ON209520	A. aculeatus	+	−	+	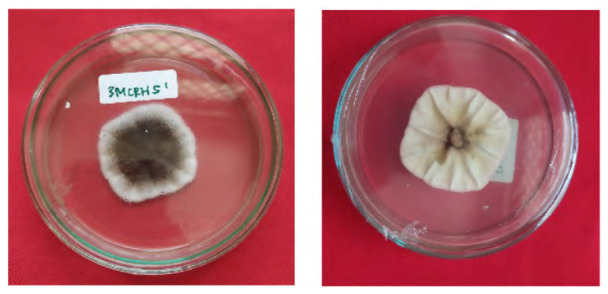
Rhizosphere	99.41	MT077159	*M. inundatum*	+	+	−	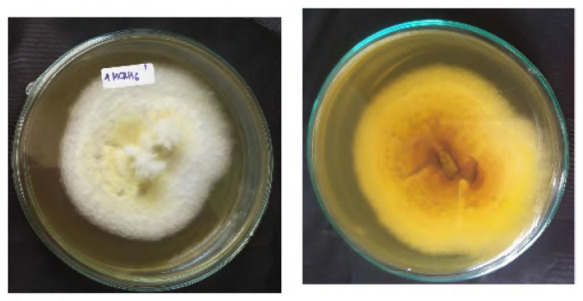
Stems	99.64	MK379959	*C. truncatum*	+	−	−	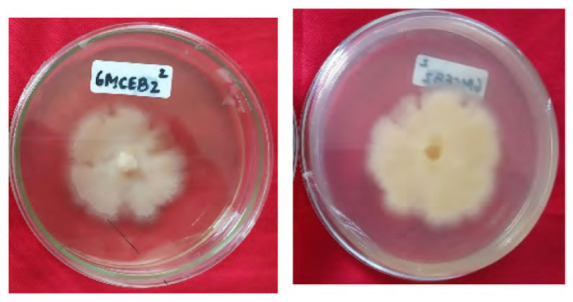

*Note*: prod. = production.
